# Never Too Much—The Benefit of Talent to Team Performance in the National Basketball Association: Comment on Swaab, Schaerer, Anicich, Ronay, and Galinsky (2014)

**DOI:** 10.1177/0956797620960396

**Published:** 2021-01-15

**Authors:** Bartosz Gula, Nemanja Vaci, Rainer W. Alexandrowicz, Merim Bilalić

**Affiliations:** 1 Department of Psychology, University of Klagenfurt; 2 Department of Psychology, University of Sheffield; 3 Department of Psychology, University of Northumbria at Newcastle

As long ago as the 4th century BCE, Aristotle (~350 BCE/1999) claimed that moderate amounts of qualities, rather than an abundance thereof, are needed for success. Indeed, there are a number of too-much-of-a-good-thing (TMGT) phenomena in psychology in which generally positive traits start to exert negative influence after a certain point (for reviews, see Grant & Schwartz, 2011; Pierce & Aguinis, 2013; for a general framework, see Busse, Mahlendorf, & Bode, 2016). Swaab, Schaerer, Anicich, Ronay, and Galinsky (2014) demonstrated such a phenomenon in team sports: Having more talented team members leads to better team performance up to a certain point, after which talent becomes “too much” and detrimental to performance. This too-much-talent (TMT) effect was present in basketball and soccer, professional team sports with high coordination requirements, presumably because status conflicts among highly skilled members impair coordination in teams. The TMT effect was absent in baseball, in which these requirements are lower. Here, we reexamine the TMT effect in basketball, the only domain in which the TMT effect has been shown,^1^ using the same data set as in the original study as well as a much larger data set. We demonstrate that Swaab et al.’s evidence of TMT is based on an inappropriate approach to testing the inverse-U-shaped relation. The results demonstrate that the common belief among laypeople (Swaab et al., 2014 Study 1) is actually correct—teams generally benefit from more talented members although the benefits decrease marginally. We did not observe any case in which increased talent was detrimental to team success.

The common approach for identifying the negative impact of talent (or any other variable) on performance is to estimate a quadratic function (Cohen, Cohen, West, & Aiken, 2003). If the quadratic function has a positive linear coefficient (which describes the initial improvement) and a negative quadratic coefficient, the latter will continuously force the fitting curve to bend and eventually continue downward. That is why it is important not only that the quadratic term is significant but also that the inflection point at which benefits turn into detriments is well within the observed talent range (see also Forster, 2000, on extrapolation error). When the data beyond the estimated inflection point (maximum of the curve) are sparse, or the true relationship toward the end of the scale is nearly flat, the approach often indicates inverse-U-shaped relations when they do not really exist (Simonsohn, 2018).

Similar problems with the quadratic function led Swaab et al. (2014) to conclude that too much talent in the National Basketball Association (NBA) is detrimental to team performance. As shown in Figure 1a, we replicated the 10 seasons of the NBA data from Swaab et al. (2014, Study 3) using their described procedure. The talent ratio (*x*-axis) indicates the proportion of the top players in a team, whereas their success (*y*-axis) is measured by the winning proportion in that particular season. The quadratic coefficient is indeed significantly negative (*b*_2_ = −1.49, *SE* = 0.61, *p* = .014), and the estimated turning point (at *xˆ* = .52) is within the observed talent range (.13–.64). However, there are only 27 data points (9% of all data) beyond the inflection point.

**Fig. 1.**
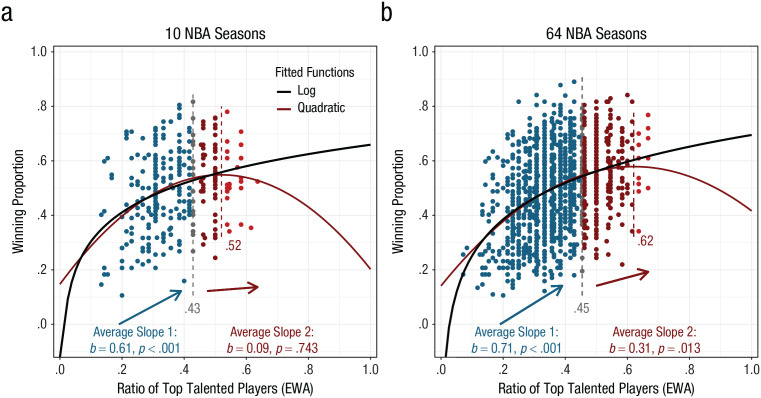
National Basketball Association (NBA) teams’ winning proportion as a function of the ratio of top talented players based on estimated wins added (EWA; Hollinger, 2009), separately for (a) 10 regular NBA seasons (2002–2012; *N* = 297; from ESPN) and (b) 64 seasons (1956–2019; *N* = 1,417; from www.basketball-reference.com). Points in blue and red represent the data corresponding to the two average slopes of the interrupted regression, and the gray dashed vertical line in between shows the break point. The red dashed vertical line shows the value of talent at the maximum of the quadratic model; points in lighter red are beyond the maximum.

The fact that the inferences are based on relatively few instances is far from ideal, and other approaches for establishing U-shaped relationships are needed. One such approach is to split the data at a breaking point and fit two straight lines, one for each part of the data (interrupted-regression approach; see Marsh & Cormier, 2001). Evidence for an inverse U shape is given if the first straight line significantly increases while the second one significantly decreases. Here, we used a procedure developed by Simonsohn (2018), which is specifically designed to improve the detection of a U shape. Figure 1a shows that even with this direct test, there is no negative effect of talent on performance in the original NBA data. As a matter of fact, the second slope, which is supposed to capture the negative relation, is flat (and not significant) rather than trending downward.

The original data are based on 10 seasons and 297 data points, which represent just a fraction of the history of a century-old game. It is possible that within these 10 seasons there are simply not enough teams with a high talent ratio, which would preclude us from reliably estimating the negative trajectory beyond the inflection point. We therefore checked whether there was a TMT effect in a larger data set spanning 64 NBA seasons from 1955–1956 to 2018–2019 (www.basketball-reference.com) and including 1,417 data points. The larger data set also illustrates the pitfalls of using a quadratic model for testing the TMT effect (Fig. 1b). The standard quadratic model has a significant negative quadratic term (*b*_2_ = −1.13, *SE* = 0.26, *p* < 0.001), indicating the inverse-U-shaped relation. The inflection point (at *xˆ* = .62) is, however, even closer to the edge of the observed talent range (.07–.67), and there are merely 13 data points beyond (less than 1% of the data). The interrupted-regression approach shows that the second line is positive and significant (*b*_2_ = 0.31, *SE* = 0.12, *p* = .013). In other words, the more talent a team has, the more successful it is, even if the positive effect diminishes over the course of the talent scale.

The analyses presented here are just a subset of the analyses we conducted on the two data sets (see the Supplemental Material available online). All other analyses demonstrate that there is little evidence for a TMT effect in the NBA domain. For example, when we use the measure of intrateam coordination instead of team performance (Swaab et al., 2014, Study 3), either coefficients for the quadratic term are not significant (for the 10-season data) or the estimated inflection point is beyond the observed talent range (64-season data; see Sections 1.4 and 2.3 in the Supplemental Material). Using different talent cutoffs does not change the results (see Section 3.2 in the Supplemental Material), nor does the inclusion of free-throw percentage, teams’ performance in the previous season, roster size, and games played as control variables (see Section 3.3 in the Supplemental Material). Separate analyses of individual periods to account for rule changes also do not support the TMT effect (see Section 3.4 in the Supplemental Material). Other established measures of player skill in the NBA (see Terner & Franks, 2020) do not show the inverse-U-shaped relation but instead depict an increase in success in relation to talent ratio (see Section 3.5 in the Supplemental Material).

Given that the quadratic function does not capture the relation between talent and team success accurately, it is fair to ask which function would be more appropriate. Obvious candidates would be functions that capture the diminishing effect talent has on success but, unlike the quadratic function, do not predict a negative relation at any point. Some of these functions, such as power, log, and logistic, describe well-known laws and principles in psychophysics (Luce, 2002; Stevens, 1957), decision making (Tversky & Kahneman, 1992), learning (Gallistel, 1990; Ruben & Wenzel, 1996), and skill acquisition (Vaci, Cocić, Gula, & Bilalić, 2019). As it turns out, one of these functions and most often the log function (black line in Fig. 1) describes the relations between talent and performance better than the quadratic function in virtually all scenarios in our analyses (see the Supplemental Material). These functions, and the log function in particular, should therefore serve as a counterpart to quadratic functions in testing for TMT effects. Not only are they the cornerstones of some of the most famous theories in psychology, but also they appropriately capture the common belief of laypeople about the relation between talent and success (Swaab et al., 2014, Study 1).

The TMGT effect is a seemingly widespread phenomenon. Be it the influence of conscientiousness on job performance (Carter et al., 2014), optimism on well-being (Milam, Richardson, Marks, Kemper, & McCutchan, 2004), or knowledge on expertise (Berman, Down, & Hill, 2002; Bilalić, McLeod, & Gobet, 2008), generally positive phenomena can exhibit negative influences after a certain point. As with TMT, most of the evidence for the broad TMGT phenomena may be based on inappropriate inferences from quadratic functions and may constitute a mere method artifact. It is possible that Aristotle and TMGT theorists were right and that too much of a certain quality is not a good thing. However, until appropriate formal tests have been used, whether interrupted regression or comparison of different functions, we should consider a never-too-much (NTM) effect.

## Supplemental Material

sj-pdf-1-pss-10.1177_0956797620960396 – Supplemental material for Never Too Much—The Benefit of Talent to Team Performance in the National Basketball Association: Comment on Swaab, Schaerer, Anicich, Ronay, and Galinsky (2014) Click here for additional data file. Supplemental material, sj-pdf-1-pss-10.1177_0956797620960396 for Never Too Much—The Benefit of Talent to Team Performance in the National Basketball Association: Comment on Swaab, Schaerer, Anicich, Ronay, and Galinsky (2014) by Bartosz Gula, Nemanja Vaci, Rainer W. Alexandrowicz and Merim Bilalić in Psychological Science

## Transparency

*Action Editor:* D. Stephen Lindsay

*Editor:* D. Stephen Lindsay

*Author Contributions*

B. Gula and M. Bilalić developed the concept for the data preparation and analyses. Data were collected and analyzed by B. Gula. B. Gula and M. Bilalić drafted the manuscript. All authors provided feedback on analyses and results and contributed critical revisions to the manuscript. All authors approved the final manuscript for submission.

**Open Practices:** Data and R code for all analyses have been made publicly available via OSF and can be accessed at https://osf.io/hfb6r. The design and analysis plans for the study were not preregistered. This article has received the badge for Open Data. More information about the Open Practices badges can be found at http://www.psychologicalscience.org/publications/badges.

## Notes

1. See the discussions by Leif Nelson and Uri Simonsohn (2014), Andrew Gelman (2016), and Roderick Swaab, Michael Schaerer, Eric Anicich, Richard Ronay, and Adam Galinsky (n.d.).
