# Supplementary material for: Never Too Much—The Benefit of Talent to Team Performance in the National Basketball Association: Comment on Swaab, Schaerer, Anicich, Ronay, and Galinsky (2014)
Source: Psychol Sci. 2021 Jan 15;32(2):301–4. doi: 10.1177/0956797620960396 (PMC7883007; doi:10.1177/0956797620960396)
Supplement: sj-pdf-1-pss-10.1177_0956797620960396 – Supplemental material for Never Too Much—The Benefit of Talent to Team Performance in the National Basketball Association: Comment on Swaab, Schaerer, Anicich, Ronay, and Galinsky (2014) [file sj-pdf-1-pss-10.1177_0956797620960396.pdf]

# Never Too Much –
# The Benefit of Talent to Team Performance in the NBA

Supplemental Online Material

Bartosz Gula, Nemanja Vaci, Rainer W. Alexandrowicz & Merim Bilalic

August, 2020

## Contents

**1 Reanalysis of 10 NBA Seasons** **2**
1.1 The ESPN Data Set . . . . . 2
1.2 Winning Proportion and Talent . . . . . 3
1.3 Outlier Analysis . . . . . 5
1.4 Intrateam Coordination and Talent . . . . . 10

**2 Analysis of 64 NBA Seasons** **12**
2.1 The Basketball-Reference Data Set . . . . . 12
2.2 Winning Proportion and Talent . . . . . 12
2.3 Intrateam Coordination and Talent . . . . . 15

**3 Generalizability Tests** **17**
3.1 LOESS Regression . . . . . 17
3.2 Different Talent Coding Ratios . . . . . 18
3.3 Inclusion of Control Variables . . . . . 19
3.4 Winning Proportion and Intrateam Coordination in different NBA periods . . . . . 22
3.5 Other Performance Measures: WS, VORP and PER . . . . . 25

First, we reanalyzed the relationship between team talent and performance (winning proportion and intrateam coordination) for the same 10 NBA seasons (ESPN data: 2002-03 to 2011-12) as reported in Swaab et al. (2014, Study 3). Player talent was defined based on estimated wins added (EWA: Hollinger, 2005). Second, in order to have a more reliable impression of the relationship, we repeated the analyses for a larger data set of 64 NBA seasons (1955-56 to 2018-19, compiled from basketball-reference.com [BR]) and included three additional definitions of talent: value over replacement player (VORP), win share (WS), and player efficiency rating (PER).

The analyses are structured as follows. We first describe the data sets and talent coding based on estimated wins added (EWA). Next, we use two approaches not used by Swaab et al. (2014) to test for the inverse u-shaped relationship: (1) interrupted regression using the Robin Hood algorithm (Simonsohn, 2018), and, (2) comarative fitting of models that can capture a change in the sign of the slope versus models that do not change in slope.

In the *interrupted regression approach*, u-shape is tested by fitting two separate lines, one for the part of the data preceding the maximum and the other for the part beyond the maximum. Evidence of an inverse u-shape is given if the slope of the first line is significant and positive and the slope of the second line is significant and negative. There are a number of procedures how to compute the critical break point between the two lines. Here we use the Robin Hood algorithm because simulation results show robustness and higher power to detect a u-shape compared to other approaches (Simonsohn, 2018). The Robin Hood algorithm

first computes the most extreme predicted y-value using a cubic spline, then an interrupted regression is computed with the median of a flat maximum region (within 1 SE of maximum y-value) as the break point, and the final break point is then set at the percentile of the flat maximum region that corresponds to the ratio of the z-values for the slope of both lines ($z_2/(z_1 + z_2)$). The last part allocates more data points to the part of the data with fewer observations in order to increase the power of the test of the corresponding slope.

In *comparative model fitting*, we estimate quadratic and cubic polynomials to capture the purported Too-Much-Talent (TMT) effect and logistic, log and power models as competitors. Whereas the two polynomials can accommodate various nonmonotonic relationships (changes in the sign of the slope), the latter models are monotonic and never predict the sign of the slope to change within the positive value ranges of talent and performance as in the present case. Logistic, log and power models are widely used in psychophysics, decision theory and learning and serve as a stronger competitor of a u-shaped relationship compared to the simple linear line in the standard approach. In order to account for model complexity, we evaluate Goodness-of-fit (GOF) based on the Bayesian Information Criterion (BIC) and the Akaike Information Criterion (AIC) (see Roberts & Pashler, 1996; Zucchini, 2000). We don't posit that any of the specific models reflects a law-like, true relationship. Instead, we use the relative GOF of the two types of models in a pragmatic sense, as indicators in favor or against the non-monotonic relationship underlying the TMT hypothesis. We analyze outliers and influential points using Mahalanobis distance, Cook's distance and DFBETA in section 1.3 where we highlight exemplarly for the 10 seasons ESPN data how too liberal criteria for Cook's distance may favor specific models. In order to see the overall consistency of results, we show model fits and parameter estimates for both approaches, separately for winning proportion and intrateam coordination.

Finally, in the *Generalizability Tests* section 3, we assess the talent to performance relationship with (1) model-free methods (LOESS: locally estimated scatterplot smoothing), (2) different ratios for talent coding, (3) control variables included, (4) separate analyses by NBA periods, and (5) different talent measures such as VORP, WS and PER.

Essentially, our analyses show that (1) the interrupted regression never indicates an inverse u-shaped relationship, (2) models reflecting no detrimental effects of talent such as log, logisitic and power mostly show a (slightly) better GOF than the quadratic model, and (3) the estimated maxima of the quadratic model are frequently at the border or beyond the observed range of talent. This overall results pattern is consistent across the 10 NBA seasons analyzed by Swaab et al (2014, Study 3), the 64 NBA seasons from BR, winning proportion and intrateam coordination, different ratios for coding talent, after accounting for control variables, different NBA periods and talent measures. Contrary to the Too-Mucht-Talent effect, we find that in general NBA teams' performance either benefits from more talent or the relationship becomes flat at higher talent levels.

Data and R code are available at https://osf.io/vz4dy/.

# 1 Reanalysis of 10 NBA Seasons

## 1.1 The ESPN Data Set

Team and player level data for the 10 regular NBA seasons 2002-03 to 2011-12 were compiled from ESPN and contain 4500 unique player level and 297 team level observations (30 teams in 7 seasons, 29 teams in 3 seasons, Charlotte excluded in the first season after founding, 2004-05). On average, 55 players (12 %) were traded within a season and were counted only once, towards the team they played the most games for (or minutes in case of ties). The data set contains 4 team-level variables (PCT - winning proportion, AST.pg - assists per game, DRB.pg - defensive rebounds per game, FGPERC.pg - field goal percentage per game), and 11 player-level variables (PLAYER, POSITION, MPG, GP, APG, FGPERC, FTPERC, DEFR, from ESPN Team Statistics, and PER_H, VA_H, EWA_H from Hollinger Player Statistics, see ESPN glossary for variable definitions).

As in Swaab et al (2014, Study 3), players were coded as talented if their Expected Wins Added (EWA) was in the top 33% of the players in a season. Team talent reflects the ratio of players on a team coded as talented. We included only those players that played at least 20% of the games for a team in a season,

resulting in 3,931 player-level observations.

## 1.2 Winning Proportion and Talent

Figure 1 shows team performance (PCT: team's winning proportion) plotted as a function of team talent for the raw data. As in the original study, we find an inverse u-shaped relationship with the standard approach: The positive linear and negative quadratic term in the quadratic model are significant ($b_1 = 1.55$, $p < .001$; $b_2 =$ -1.49, $p = 0.014$). The model estimates a maximum PCT of 0.55 at a talent ratio of 0.52 ($-b_1/(2{\times}b_2)$), well within the observed value range (0.13 – 0.64). There are 27 cases (less than 9 % of the data) beyond the maximum. The parameter estimates are similar to those reported by Swaab et al. (2014, p. 1586). We assume that discrepancies mainly result from different handling of traded players.

The interrupted regression estimates the break point at 0.43. The first slope is positive ($b_1 = 0.61$, $p < .001$) and the second slope is not significant ($b_2 = 0.09$, $p = .743$), suggesting no evidence for the inverse u-shape underlying the TMT hypothesis. Comparative model fitting shows that GOF measures BIC and AIC are similar, with the BIC favoring the log (BIC = -299.93) over the quadratic model (BIC = -296.47, see GOF table below). Differences between the log and the quadratic model are more pronounced at the edges and beyond the observed value range. The fitted models in Fig. 1 are extrapolated beyond the observed value range in order to visualize the models' overall shape. We do not suggest that the captured relationship extends beyond the observed range (see Forster, 2000 on extrapolation error).

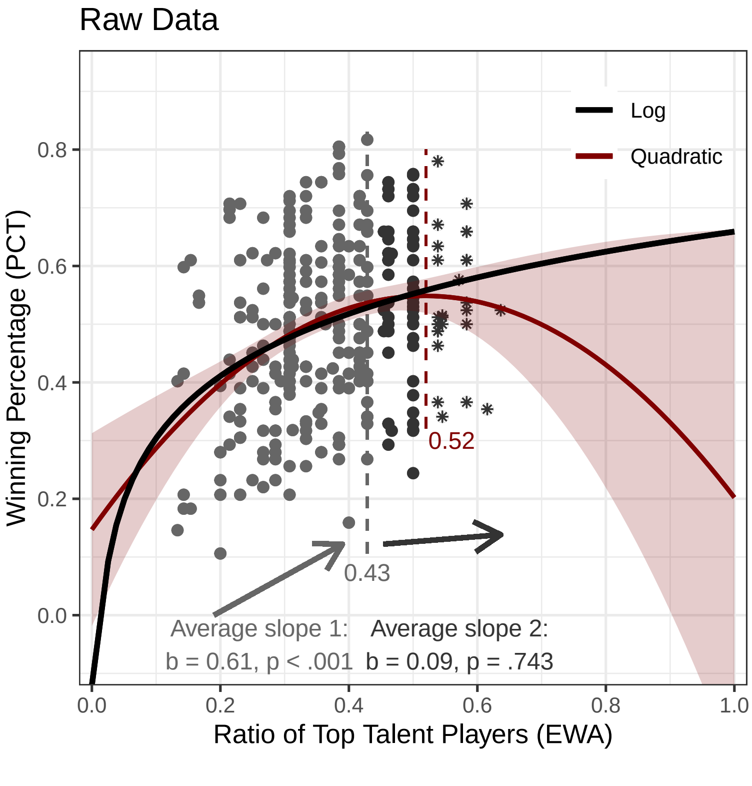


Figure 1: Fitted quadratic model (red), interrupted regression (indicated by the two gray arrows) and log model (black) for the raw data. The vertical gray line shows the break point determined by the RH algorithm, and the points in light/dark gray on either side represent the data corresponding to the two slopes of the interrupted regression. The red vertical line shows the value of Talent at the estimated maximum of the quadratic model. Stars refer to data points beyond the maximum. Red shaded area shows the 95% confidence region for the quadratic model. The figure corresponds to Fig 1 (A) in the main text.

Table 1: Goodness-of-Fit for six models.

| | LogLik | BIC | AIC | df | $R^2$ | p [$\Delta R^2$] |
|---|---|---|---|---|---|---|
| Logistic | 157.21 | -297.33 | -308.41 | 3 | - | - |
| Power | 158.04 | -298.99 | -310.07 | 3 | - | - |
| Log | 158.51 | -299.93 | -311.01 | 3 | 0.10 | - |
| Linear | 156.58 | -296.07 | -307.15 | 3 | 0.09 | - |
| Quadratic | 159.62 | -296.47 | -311.25 | 4 | 0.11 | 0.01 |
| Cubic | 159.72 | -290.97 | -309.44 | 5 | 0.11 | 0.66 |

Table 2: Parameter estimates for all seven models.

| | | Estimate | Std. Error | t/z values | p |
|---|---|---|---|---|---|
| Quadratic Model | | | | | |
| | b0 | 0.147 | 0.084 | 1.746 | 0.082 |
| | b1 | 1.549 | 0.461 | 3.358 | <.001 |
| | b2 | -1.494 | 0.605 | -2.470 | 0.014 |
| Two Lines Regression | | | | | |
| | b1 | 0.610 | 0.148 | 4.135 | <.001 |
| | b2 | 0.089 | 0.271 | 0.328 | 0.743 |
| | rh.breakpoint | 0.429 | . | . | . |
| Linear Model | | | | | |
| | b0 | 0.341 | 0.030 | 11.233 | <.001 |
| | b1 | 0.426 | 0.078 | 5.474 | <.001 |
| Logistic Model | | | | | |
| | b0 | -0.190 | 0.042 | -4.547 | <.001 |
| | b1 | 2.171 | 0.530 | 4.096 | <.001 |
| Power Model | | | | | |
| | b1 | 0.685 | 0.039 | 17.501 | <.001 |
| | b2 | 0.309 | 0.056 | 5.471 | <.001 |
| Log Model | | | | | |
| | b0 | 0.659 | 0.028 | 23.315 | <.001 |
| | b1 | 0.154 | 0.026 | 5.849 | <.001 |
| Cubic Model | | | | | |
| | b0 | 0.227 | 0.202 | 1.124 | 0.262 |
| | b1 | 0.793 | 1.792 | 0.442 | 0.659 |
| | b2 | 0.689 | 5.031 | 0.137 | 0.891 |
| | b3 | -1.958 | 4.480 | -0.437 | 0.662 |

*Note:*

Quadratic Model: Y = b0 + b1 * X + b2 *X^2

Two Lines Regression: Y = a + b1 * X_low + b2 * X_high + d*high

Linear Model: Y = b0 + b1 * X

Logistic Model: Y = b0 + 1 / (1 + e^(-b1 * X))

Power Model: Y = b1 * X^b2

Log Model: Y = b0 + b1 * log(X)

Cubic Model: Y = b0 + b1 * X + b2 * X^2 + b3 * X^3

t/z values contains z-values for interrupted regression, t-values for all others.

## 1.3 Outlier Analysis

We checked for three types of outliers: (1) univariate outliers based on a standard boxplot (i.e. obs 1.5 × interquartile range above 3rd or below 1st quartile), (2) bivariate outliers based on Mahalanobis-MCD distance (Rousseeuw & Van Zomeren, 1990), and (3) influential points based on Cook's distance (used in Swaab et al., 2014) and DFBETA2 for the coefficient of the quadratic term (c.f. Cohen, Cohen, West, & Aiken, 2003; Tabachnick & Fidell, 2014). The definitions of observations as extreme underlying the distance measures are quite different. Mahalanobis $d$ is a measure related to leverage and gives the distance of each data point from the centroid of the sample. Cook's $d$ in contrast, is a measure of influence and reflects the aggregate change in weights of a model when the point is removed (global influence). DFBETA is a measure of specific influence that quantifies for each observation how each coefficient in the regression model would change if the observation was included. In the present case DFBETA2 denotes the corresponding change for the quadratic coefficient. A positive sign implies that the coefficent is increased after inclusion (here reducing the size of the negative quadratic effect), a negative sign means decrease. Negative values thus imply that the point contributes in favor of the TMT effect, and positive values against it, with the size of DFBETA2 reflecting the magnitude of the contribution.

The data contains neither univariate outliers nor extreme Mahalanobis distances. Cook's distance with the critical cutoff of $4/N = .0135$ (used in Swaab et al.) suggest 16 cases listed in Table 3. The distance measures with common cutoff values are plotted in Figure 2. For Cook's d the rather liberal $4/N$ cutoff ($d_{crit} = 0.013$) is shown. More conservative criteria from the literature (e.g. Cohen, Cohen, West, & Aiken, 2003) suggest to set the cutoff at $F(2, 295)$ and $\alpha = .50$, which in the present case would not flag any case as extreme (with $d_{crit} = 0.69$). The DFBETA2 values are mostly negative which implies that removing them would contribute against the TMT hypothesis.

Table 3: Outliers identified by Cook's d (and a linear model) with the 4/N criterion.

| | SEASON | TEAM | PCT | TALENT | cook.d1 | mahal.d1 | DFBETA2 |
|---|---|---|---|---|---|---|---|
| 28 | 2009-10 | Brooklyn | 0.146 | 0.1333 | 0.0332 | 8.753 | -0.275 |
| 94 | 2008-09 | Golden State | 0.354 | 0.6154 | 0.0321 | 8.412 | -0.265 |
| 37 | 2011-12 | Charlotte | 0.106 | 0.2000 | 0.0318 | 8.279 | -0.175 |
| 175 | 2009-10 | Minnesota | 0.183 | 0.1429 | 0.0234 | 7.517 | -0.210 |
| 153 | 2007-08 | Miami | 0.183 | 0.1538 | 0.0225 | 7.176 | -0.199 |
| 169 | 2003-04 | Minnesota | 0.707 | 0.2143 | 0.0207 | 5.988 | 0.133 |
| 160 | 2004-05 | Milwaukee | 0.366 | 0.5833 | 0.0203 | 6.502 | -0.173 |
| 157 | 2011-12 | Miami | 0.697 | 0.2143 | 0.0192 | 5.720 | 0.129 |
| 203 | 2007-08 | Oklahoma City | 0.244 | 0.5000 | 0.0189 | 6.429 | -0.037 |
| 184 | 2008-09 | New Orleans | 0.598 | 0.1429 | 0.0188 | 6.663 | 0.354 |
| 68 | 2002-03 | Denver | 0.207 | 0.1429 | 0.0186 | 6.987 | -0.178 |
| 128 | 2002-03 | LA Lakers | 0.610 | 0.1538 | 0.0186 | 6.365 | 0.310 |
| 263 | 2007-08 | San Antonio | 0.683 | 0.2143 | 0.0172 | 5.361 | 0.123 |
| 156 | 2010-11 | Miami | 0.707 | 0.2308 | 0.0169 | 5.351 | 0.083 |
| 138 | 2002-03 | Memphis | 0.341 | 0.5455 | 0.0161 | 5.438 | -0.109 |
| 176 | 2010-11 | Minnesota | 0.207 | 0.2000 | 0.0149 | 5.408 | -0.114 |

Figure 3 shows the results of the quadratic model fit and interrupted regression if the $4/N$ cutoff is applied, using either a quadratic or linear model for Cook's d. Using a quadratic model, 17 observations are removed. The size of the estimate of the quadratic term is larger compared to the raw data analysis ($b_1 = 1.98$, $SE = 0.57$, $p = <.001$, $b_2 = -1.96$, $SE = 0.73$, $p = 0.007$). The estimated maximum of the quadratic model is moved to the left and lies at a talent ratio of 0.51 (and PCT = 0.55, talent range: 0.13 - 0.64). Using a linear model for Cook's d (16 obs removed), the size of the estimate for the quadratic term becomes insignificant ($b_1 = 1.32$, $SE = 0.54$, $p = 0.015$, $b_2 = -1.09$, $SE = 0.69$, $p = 0.115$) and the estimated maximum of the quadratic model is moved to the right at a talent ratio of 0.6 (and PCT = 0.57, talent range: 0.13 - 0.64).

Figure 3 illustrates that the two ways to compute Cook's distance paired with a liberal cut-off introduce bias

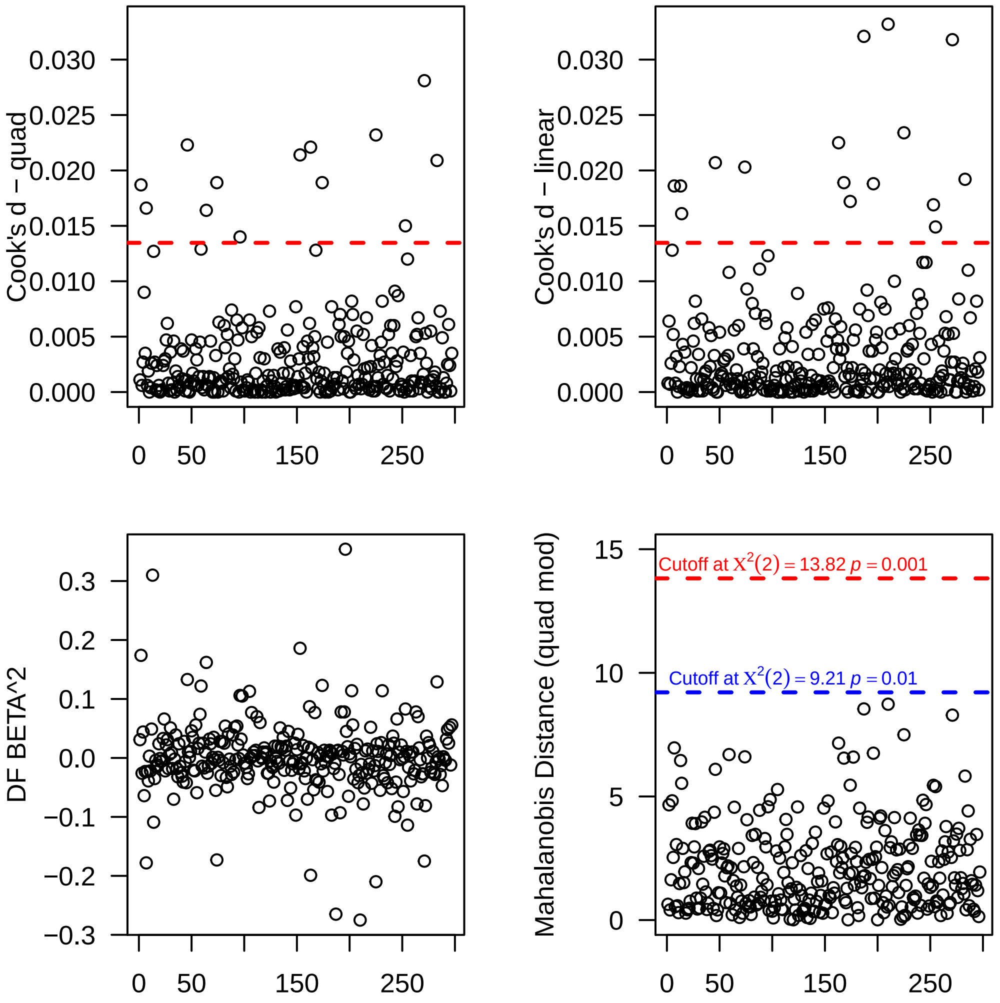


Figure 2: Outlier measures: Cook's distance in top panels, based on quadratic (left) and linear model (right). Mahalanobis distance and DFBETA2 values in bottom panels. Horizontal dashed lines show critical cutoffs.

when testing hypotheses about the talent performance relationship. Assuming a quadratic model for Cook's d will favor the TMT hypothesis and assuming a linear model will provide evidence against it. In order to exclude plausible outliers in a way that minimizes any selective advantage given to a specific model, we therefore used the following criteria in all subsequent analyses. Univariate outliers based on Tukey's boxplot statistics were removed first (see Tabachnick & Fidell, 2014). Next, bivariate outliers were removed if either Mahalanobis Distance (MCD) was larger than $d_{crit} = \chi^2(2) = 13.82, p = .001$, or Cook's distance based on the linear and the quadratic model were *both* larger than $F(2, N-2)$ at $\alpha = .50$. These more conservative criteria generally identify fewer observations as extreme compared to the $4/N$ criterion used in Swaab et al. (2014).

For transparency, we report the GOF results from comparative model fitting and parameter estimates after outliers were exluded according to the liberal criteria, separateley for Cook's d based on the linear and quadratic model at the end of the section (Tables 4-7). Although the type of outlier exclusion affects the location of the estimated maximum, the general pattern of results for interrupted regression and comparative model fitting is still similar. The 2nd slope is nonsigifnificant, and the log, logistic and power models fit better than the quadratic model according to BIC.

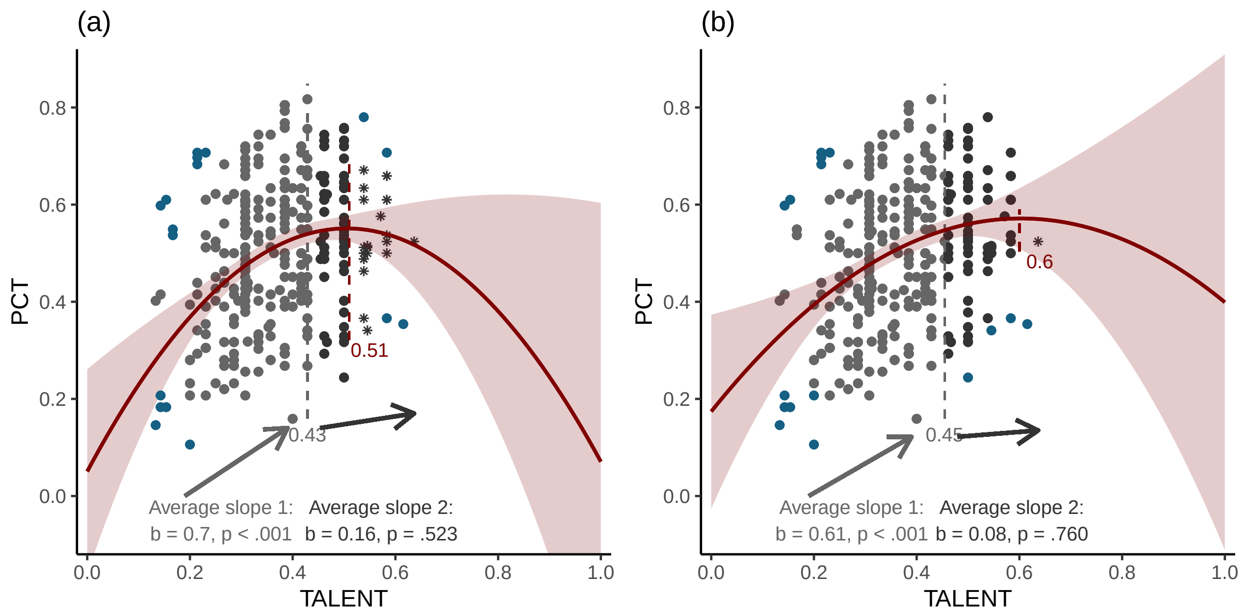


Figure 3: Fits of the quadratic model when outliers (marked blue) are removed. Panel (a) shows outliers from Cook's distance based on a quadratic model, panel (b) shows outliers based on a linear model (cutoff: 4/N).

Table 4: Goodness-of-Fit for six models after exclusion of outliers (Cook's d, linear model).

| | LogLik | BIC | AIC | df | $R^2$ | p [$\Delta R^2$] |
|---|---|---|---|---|---|---|
| Logistic | 165.73 | -314.55 | -325.46 | 3 | - | - |
| Power | 165.88 | -314.85 | -325.76 | 3 | - | - |
| Log | 165.99 | -315.06 | -325.98 | 3 | 0.11 | - |
| Linear | 165.21 | -313.50 | -324.42 | 3 | 0.11 | - |
| Quadratic | 166.46 | -310.37 | -324.93 | 4 | 0.12 | 0.12 |
| Cubic | 166.61 | -305.02 | -323.21 | 5 | 0.12 | 0.60 |

Table 5: Parameter estimates for all seven models with exclusion based on Cook's with from a linear model.

| | | Estimate | Std. Error | t/z values | p |
|---|---|---|---|---|---|
| Quadratic Model | | | | | |
| | b0 | 0.174 | 0.101 | 1.715 | 0.087 |
| | b1 | 1.319 | 0.540 | 2.441 | 0.015 |
| | b2 | -1.094 | 0.692 | -1.580 | 0.115 |
| Two Lines Regression | | | | | |
| | b1 | 0.610 | 0.133 | 4.585 | <.001 |
| | b2 | 0.084 | 0.275 | 0.305 | 0.760 |
| | rh.breakpoint | 0.454 | . | . | . |
| Linear Model | | | | | |
| | b0 | 0.326 | 0.032 | 10.196 | <.001 |
| | b1 | 0.475 | 0.081 | 5.843 | <.001 |
| Logistic Model | | | | | |
| | b0 | -0.215 | 0.053 | -4.080 | <.001 |
| | b1 | 2.546 | 0.712 | 3.577 | <.001 |
| Power Model | | | | | |
| | b1 | 0.715 | 0.042 | 16.847 | <.001 |
| | b2 | 0.348 | 0.060 | 5.782 | <.001 |
| Log Model | | | | | |
| | b0 | 0.681 | 0.030 | 22.622 | <.001 |
| | b1 | 0.173 | 0.029 | 5.990 | <.001 |
| Cubic Model | | | | | |
| | b0 | 0.302 | 0.262 | 1.154 | 0.250 |
| | b1 | 0.186 | 2.203 | 0.085 | 0.933 |
| | b2 | 2.043 | 5.956 | 0.343 | 0.732 |
| | b3 | -2.740 | 5.167 | -0.530 | 0.596 |

*Note:*
Quadratic Model: Y = b0 + b1 * X + b2 *X^2
Two Lines Regression: Y = a + b1 * X_low + b2 * X_high + d*high
Linear Model: Y = b0 + b1 * X
Logistic Model: Y = b0 + 1 / (1 + e^(-b1 * X))
Power Model: Y = b1 * X^b2
Log Model: Y = b0 + b1 * log(X)
Cubic Model: Y = b0 + b1 * X + b2 * X^2 + b3 * X^3
t/z values contains z-values for interrupted regression, t-values for all others.

Table 6: Goodness-of-Fit for six models after exclusion of outliers (Cook's d, quad model).

| | LogLik | BIC | AIC | df | $R^2$ | p [$\Delta R^2$] |
|---|---|---|---|---|---|---|
| Logistic | 164.26 | -311.62 | -322.53 | 3 | - | - |
| Power | 162.43 | -307.96 | -318.87 | 3 | - | - |
| Log | 163.02 | -309.13 | -320.03 | 3 | 0.11 | - |
| Linear | 161.06 | -305.21 | -316.12 | 3 | 0.10 | - |
| Quadratic | 164.70 | -306.87 | -321.40 | 4 | 0.12 | 0.01 |
| Cubic | 164.71 | -301.24 | -319.41 | 5 | 0.12 | 0.93 |

Table 7: Parameter estimates for all seven models with exclusion based on Cook's with from a quadratic model.

| | | Estimate | Std. Error | t/z values | p |
|---|---|---|---|---|---|
| Quadratic Model | | | | | |
| | b0 | 0.051 | 0.107 | 0.473 | 0.637 |
| | b1 | 1.981 | 0.568 | 3.488 | <.001 |
| | b2 | -1.961 | 0.726 | -2.702 | 0.007 |
| Two Lines Regression | | | | | |
| | b1 | 0.700 | 0.140 | 4.993 | <.001 |
| | b2 | 0.164 | 0.256 | 0.639 | 0.523 |
| | rh.breakpoint | 0.429 | . | . | . |
| Linear Model | | | | | |
| | b0 | 0.326 | 0.033 | 9.898 | <.001 |
| | b1 | 0.463 | 0.084 | 5.538 | <.001 |
| Logistic Model | | | | | |
| | b0 | -0.419 | 0.030 | -14.059 | <.001 |
| | b1 | 6.917 | 1.229 | 5.626 | <.001 |
| Power Model | | | | | |
| | b1 | 0.708 | 0.044 | 16.265 | <.001 |
| | b2 | 0.347 | 0.062 | 5.562 | <.001 |
| Log Model | | | | | |
| | b0 | 0.679 | 0.031 | 21.952 | <.001 |
| | b1 | 0.177 | 0.030 | 5.917 | <.001 |
| Cubic Model | | | | | |
| | b0 | 0.029 | 0.282 | 0.103 | 0.918 |
| | b1 | 2.170 | 2.351 | 0.923 | 0.357 |
| | b2 | -2.478 | 6.303 | -0.393 | 0.694 |
| | b3 | 0.449 | 5.435 | 0.083 | 0.934 |

*Note:*
Quadratic Model: Y = b0 + b1 * X + b2 *X^2
Two Lines Regression: Y = a + b1 * X_low + b2 * X_high + d*high
Linear Model: Y = b0 + b1 * X
Logistic Model: Y = b0 + 1 / (1 + e^(-b1 * X))
Power Model: Y = b1 * X^b2
Log Model: Y = b0 + b1 * log(X)
Cubic Model: Y = b0 + b1 * X + b2 * X^2 + b3 * X^3
t/z values contains z-values for interrupted regression, t-values for all others.

## 1.4 Intrateam Coordination and Talent

Intrateam coordination was computed same as in Swaab et al. (2014), as an index measure from (1) field goal percentage (FGPERC.pg), (2) average number of defensive rebounds per game (DRB.pg), and (3) average number of assists per game (AST.pg). The team-level measures were then standardized (for each season) and averaged. In Figure 4 we show the fit of the quadratic model, the slopes of the interrupted regression, and the best fitting model according to BIC, separately for the raw data (panel a) and after exclusion of outliers based on the procedure described above (panel B).

Our outlier procedure identifies four univariate outliers, the distance measures don't flag any of the remaining data points as extreme (maximum Cook's $d$ (quadratic) = 0.107; maximum Mahalanobis $d$ = 11.166). After exclusion of the univariate outliers, we find an insignificant quadratic term for the quadratic model ($b_2$ = -3.18, $p$ = 0.278), the talent ratio 0.68 corresponding to the estimated PCT maximum is beyond the observed value range (0.13 – 0.64). The interrupted regression has an insignificant second slope ($b_2$ = 1.26, $p$ = 0.530). The Log model fits the data better than the Quadratic model ($\text{BIC}_{log}$ = 625.17 vs. $\text{BIC}_{quad}$ = 630.70). Please note that we estimated the power model with an intercept $b_0$, and added the $b_1$ parameter to the logistic model. The simpler versions of the models used in the anlaysis of PCT fitted the data worse. The GOF and parameter estimates are listed below.

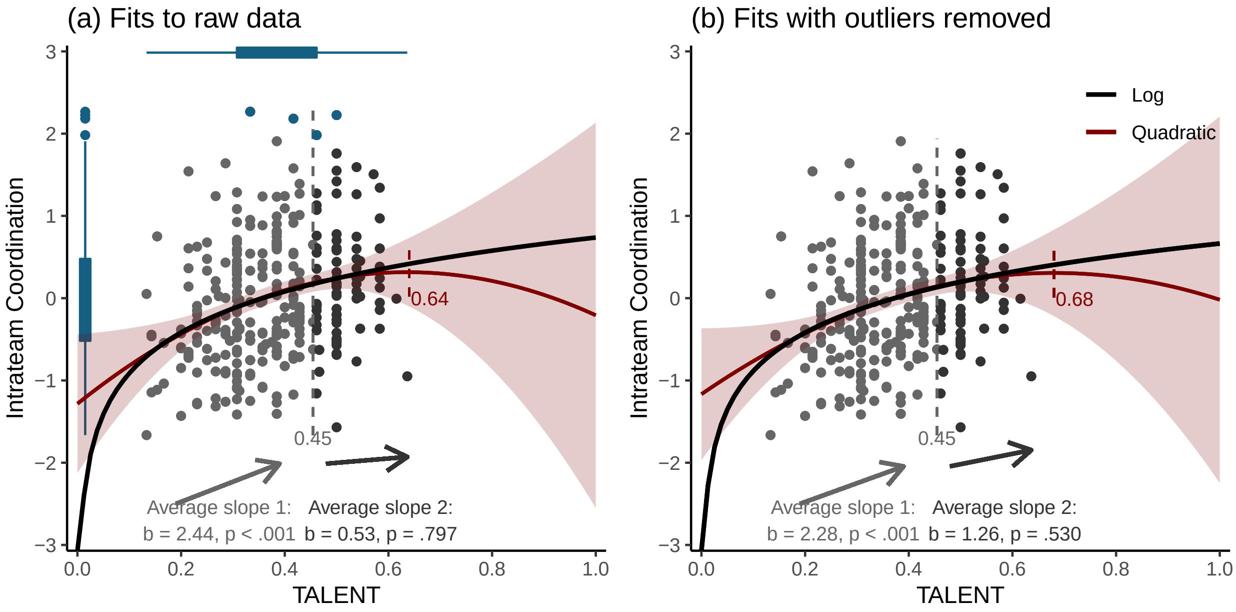


Figure 4: Intrateam coordination and Talent. Fits of the quadratic model, log model and slopes of interrupted regression are shown. Break point of interrupted regression is shown as gray vertical line and talent at maximum of quadratic model is marked red. Panel (a) shows fits for raw data and panel (b) for data without outliers. Boxplots show univariate outliers.

Table 8: Goodness-of-Fit for six models.

| | LogLik | BIC | AIC | df | $R^2$ | p [$\Delta R^2$] |
|---|---|---|---|---|---|---|
| Logistic | -308.22 | 639.17 | 624.45 | 4 | - | - |
| Power | -304.37 | 631.45 | 616.73 | 4 | - | - |
| Log | -304.07 | 625.17 | 614.13 | 3 | 0.09 | - |
| Linear | -304.58 | 626.21 | 615.17 | 3 | 0.08 | - |
| Quadratic | -303.99 | 630.70 | 615.98 | 4 | 0.09 | 0.28 |
| Cubic | -303.92 | 636.23 | 617.83 | 5 | 0.09 | 0.70 |

Table 9: Parameter estimates for all seven models.

| | | Estimate | Std. Error | t/z values | p |
|---|---|---|---|---|---|
| Quadratic Model | | | | | |
| | b0 | -1.167 | 0.407 | -2.866 | 0.004 |
| | b1 | 4.333 | 2.234 | 1.940 | 0.053 |
| | b2 | -3.185 | 2.929 | -1.087 | 0.278 |
| Two Lines Regression | | | | | |
| | b1 | 2.277 | 0.601 | 3.785 | <.001 |
| | b2 | 1.263 | 2.010 | 0.628 | 0.530 |
| | rh.breakpoint | 0.454 | . | . | . |
| Linear Model | | | | | |
| | b0 | -0.754 | 0.146 | -5.168 | <.001 |
| | b1 | 1.939 | 0.374 | 5.178 | <.001 |
| Logistic Model | | | | | |
| | b1 | -2.420 | 0.945 | -2.561 | 0.011 |
| | b2 | 2.502 | 0.965 | 2.592 | 0.010 |
| | b3 | 8.835 | 2.776 | 3.182 | 0.002 |
| | b4 | 0.724 | 0.033 | 21.813 | <.001 |
| Power Model | | | | | |
| | b0 | -1.004 | 0.645 | -1.556 | 0.121 |
| | b1 | 1.978 | 0.511 | 3.873 | <.001 |
| | b2 | 0.708 | 0.480 | 1.473 | 0.142 |
| | b3 | 0.690 | 0.029 | 23.850 | <.001 |
| Log Model | | | | | |
| | b0 | 0.664 | 0.137 | 4.853 | <.001 |
| | b1 | 0.673 | 0.127 | 5.286 | <.001 |
| Cubic Model | | | | | |
| | b0 | -0.829 | 0.980 | -0.846 | 0.398 |
| | b1 | 1.144 | 8.688 | 0.132 | 0.895 |
| | b2 | 6.016 | 24.398 | 0.247 | 0.805 |
| | b3 | -8.254 | 21.728 | -0.380 | 0.704 |

*Note:*
Quadratic Model: Y = b0 + b1 * X + b2 *X^2
Two Lines Regression: Y = a + b1 * X__low + b2 * X__high + d*high
Linear Model: Y = b0 + b1 * X
Logistic Model: Y = b0 + b1 / (1 + e^(-b2 * X))
Power Model: Y = b0 + b1 * X^b2
Log Model: Y = b0 + b1 * log(X)
Cubic Model: Y = b0 + b1 * X + b2 * X^2 + b3 * X^3
t/z values contains z-values for interrupted regression, t-values for all others.

## 2 Analysis of 64 NBA Seasons

### 2.1 The Basketball-Reference Data Set

If there was a maximum of talent ratio beyond which more talent was worse for the team, and especially if such a maximum was close to the border of the observed talent ratio range, then a better data setting would include more observations at the upper end of the talent ratio range. Therefore, we compiled a data set from basketball-reference.com (BR) that spans 64 NBA seasons (1955-56 to 2018-19) and contains 20,556 player level observations. A similar approach to data preparation was used as before. The data starts with the 10th NBA season (1955-56), because the 24-seconds shot clock rule was introduced in the season before, and the 8 NBA franchises in that season (and all franchises founded afterwards) are still active today. Historic team names were changed to current franchise names (e.g. "St. Louis Hawks" to "Atlanta Hawks"). The data set (br_dat) contains 50 variables, the first 25 vars are team level info and stats compiled mainly from Franchise Totals or Team Totals. Variables 26-50 contain player level statistics from each Team's Season Stats (per game and advanced, e.g. Boston Celtics 1956, see the BR glossary pages for variable definitions).

BR does not provide EWA, which was computed from PER based on the ESPN formula (Hollinger, 2005). Traded players were counted once in a season, towards the team they played the most games for. Their EWA was computed from their average PER across all teams they played for, weighted by minutes played. For the 10 seasons that overlap between the ESPN and BR player-level EWA and PER correlated strongly ($r = .951$ for EWA, and $r = .997$ for PER).

Again, players were coded as talented if their EWA was in the top 33% of players in a season and team talent was calculated as the ratio of talented players. Only 18,073 players who played 20% or more of the games for a team in a season were included, resulting in 1,417 team level observations.

### 2.2 Winning Proportion and Talent

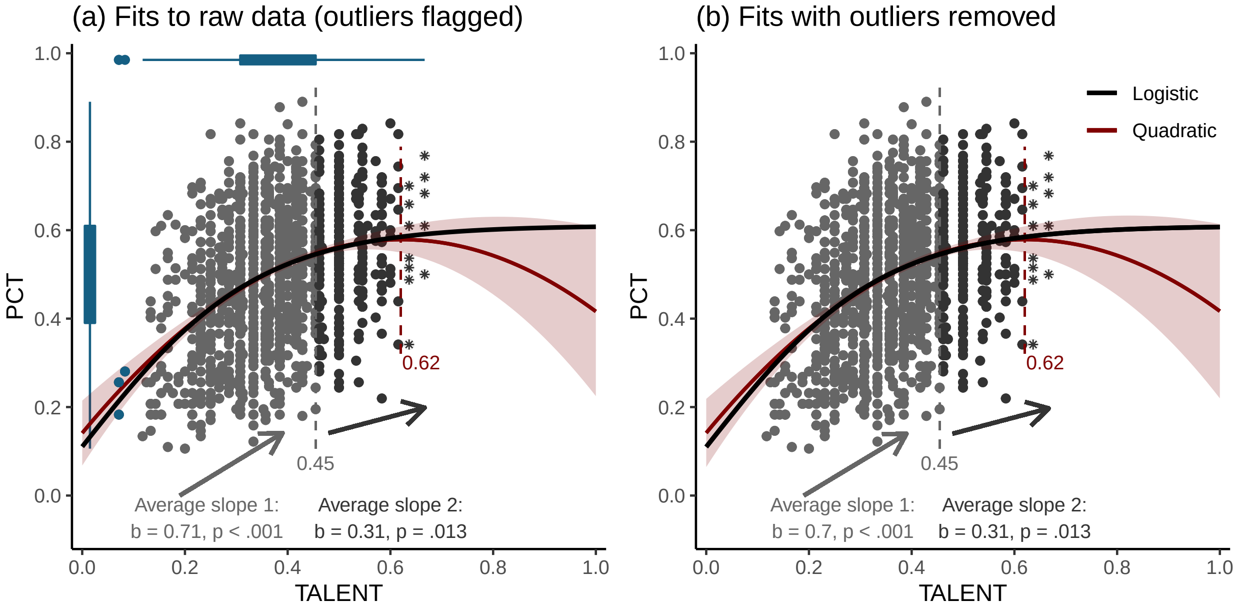


Figure 5: Winning proportion and Talent. Fits of quadratic model, log model and slopes of interrupted regression are shown. Break point of interrupted regression is shown as gray vertical line, and talent at maximum of quadratic model in red. Panel (a) shows fits for raw data and panel (b) for data without outliers. Red shaded area shows the 95% confidence region for the quadratic model. Left figure corresponds to Fig 1 (B) in the main text.

Our outlier procedure identifies only 3 univariate outliers, the distance measures don't flag any data points

as extreme (maximum Cook's $d$ (linear) = 0.014; maximum Mahalanobis $d$ = 12.58). For two of the three univariate outliers in the bottom left segment (see Figure 5 (a)) it was the inaugural season (New Orleans Jazz in 1974-75 and Miami Heat in 1988-89). In the 3rd case (Chicago Bulls in 2001-02), the Bulls lost several top scorers in midseason (by trading Ron Artest, Brad Miller and Ron Mercer to Indiana), which Bulls fans discuss as one of the worst trading decisions in the teams history [1].

After exclusion, we find significant coefficients for the quadratic model ($b_1$ = 1.41, $p$ = <.001, $b_2$ = -1.13, $p$ = <.001), and the talent ratio 0.622 corresponding to the estimated maximum PCT is within the observed value range (0.118 – 0.667). The standard approach thus supports the TMT hypothesis, although the log-based model is within the confidence limits of the quadratic model across the entire range. Moreover, within the observed range of TALENT, the quadratic curve nestles closely to the log-curve (in fact, they are barely distinguishable. In fact, the major difference appears in a region without data (TALENT > 0.8). From that point of view, the TMT-effect might emerge as a pure methods artifact. Only 13 cases (less than 1 % of the data) are beyond the maximum, and more than half of these points actually has a higher PCT than the max PCT estimated by the quadratic model.

The RH algorithm of the interrupted regression determines the breakpoint at 0.45, the 2nd slope is significant and positive ($b_2$ = 0.31, $p$ = 0.013). Comparative model fitting shows that all three monotonic models (log, logistic and power) provide a better GOF than the quadratic and cubic models, best fit is found for the logistic model (BIC$_{logi}$ = -1548.46 vs. BIC$_{quad}$ = -1540.56). See below for the overall GOF results and model estimates. The results from both approaches show no evidence of a TMT effect, rather teams benefit from more talented players. Given the relatively worst fit for the linear model, the benefit is likely marginally decreasing. The interrupted regression can be checked via Simonsohn's web-app (http://webstimate.org/twolines/) by uploading the br_data1.csv file we provide on osf (PCT ~TALENT).

Table 10: Goodness-of-Fit for six models.

| | LogLik | BIC | AIC | df | $R^2$ | p [$\Delta R^2$] |
|---|---|---|---|---|---|---|
| Logistic | 785.11 | -1548.46 | -1564.22 | 3 | - | - |
| Power | 781.84 | -1541.91 | -1557.67 | 3 | - | - |
| Log | 784.11 | -1546.45 | -1562.21 | 3 | 0.14 | - |
| Linear | 775.78 | -1529.80 | -1545.57 | 3 | 0.13 | - |
| Quadratic | 784.79 | -1540.55 | -1561.57 | 4 | 0.14 | 0.0 |
| Cubic | 785.14 | -1534.01 | -1560.28 | 5 | 0.14 | 0.4 |

Table 11: Parameter estimates for all seven models.

| | | Estimate | Std. Error | t/z values | p |
|---|---|---|---|---|---|
| Quadratic Model | | | | | |
| | b0 | 0.142 | 0.039 | 3.604 | <.001 |
| | b1 | 1.407 | 0.208 | 6.764 | <.001 |
| | b2 | -1.132 | 0.266 | -4.252 | <.001 |
| Two Lines Regression | | | | | |
| | b1 | 0.699 | 0.059 | 11.923 | <.001 |
| | b2 | 0.307 | 0.124 | 2.478 | 0.013 |
| | rh.breakpoint | 0.454 | . | . | . |
| Linear Model | | | | | |
| | b0 | 0.297 | 0.014 | 20.817 | <.001 |
| | b1 | 0.536 | 0.036 | 14.746 | <.001 |
| Logistic Model | | | | | |
| | b0 | -0.390 | 0.020 | -19.424 | <.001 |
| | b1 | 5.886 | 0.626 | 9.397 | <.001 |
| Power Model | | | | | |
| | b1 | 0.740 | 0.020 | 37.273 | <.001 |
| | b2 | 0.394 | 0.027 | 14.458 | <.001 |
| Log Model | | | | | |
| | b0 | 0.697 | 0.013 | 52.510 | <.001 |
| | b1 | 0.194 | 0.013 | 15.386 | <.001 |
| Cubic Model | | | | | |
| | b0 | 0.070 | 0.094 | 0.748 | 0.455 |
| | b1 | 2.049 | 0.793 | 2.585 | 0.010 |
| | b2 | -2.907 | 2.132 | -1.364 | 0.173 |
| | b3 | 1.531 | 1.824 | 0.839 | 0.401 |

*Note:*
Quadratic Model: Y = b0 + b1 * X + b2 *X^2
Two Lines Regression: Y = a + b1 * X_low + b2 * X_high + d*high
Linear Model: Y = b0 + b1 * X
Logistic Model: Y = b0 + 1 / (1 + e^(-b1 * X))
Power Model: Y = b1 * X^b2
Log Model: Y = b0 + b1 * log(X)
Cubic Model: Y = b0 + b1 * X + b2 * X^2 + b3 * X^3
t/z values contains z-values for interrupted regression, t-values for all others.

## 2.3 Intrateam Coordination and Talent

Intrateam coordination was computed as before. In Figure 6 we show the fit of the quadratic model, the slopes of the interrupted regression, and the best fitting model according to BIC, separately for the raw data and after exclusion of outliers based on the procedure described in Section 1.3.

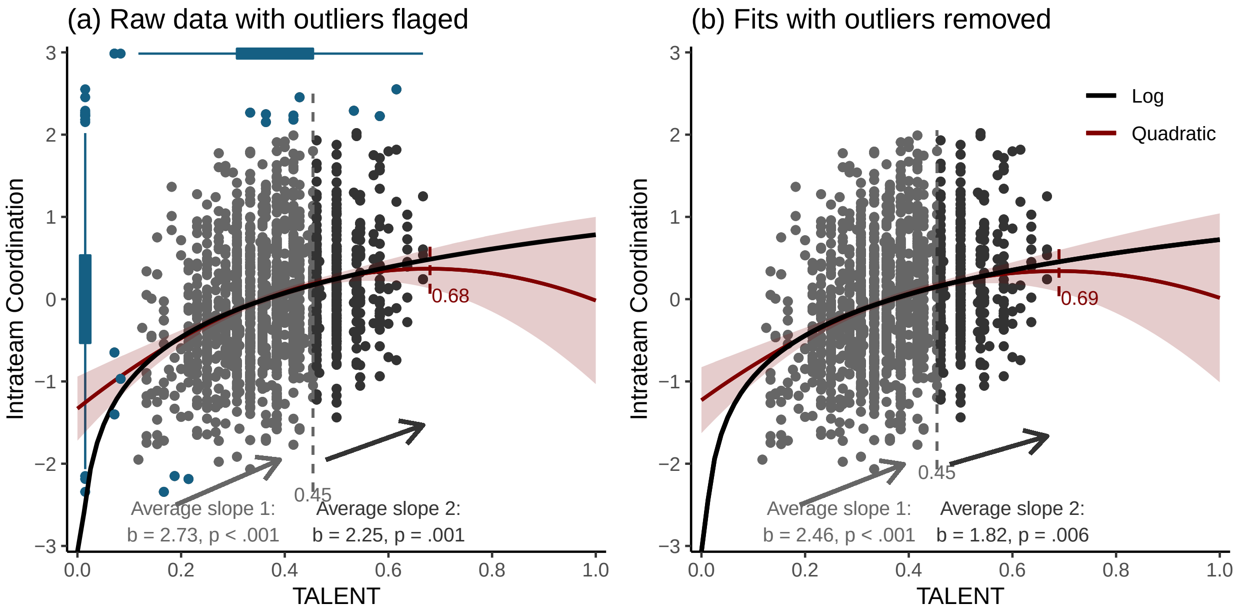


Figure 6: Intrateam coordination and Talent. Fits of the quadratic model, log model and slopes of interrupted regression are shown. Panel (a) shows fits for raw data and panel (b) for data without outliers.

After exclusion of 15 univariate outliers (no extreme distance measures: maximum Cook's $d$ (linear) = 0.011, maximum Mahalanobis $d = 11.44$), we find significant coefficients for the quadratic model ($b_1 = 4.57$, $p =$ 4.219, $b_2 =$ -3.33, $p =$ -2.400). However, the talent ratio 0.687 corresponding to the estimated maximum PCT is beyond the observed value range (0.118 – 0.667).

The RH algorithm of the interrupted regression determines the breakpoint at 0.45, the 2nd slope is significant and positive ($b_2 = 1.82$, $p = 0.006$). Compartative model fitting shows that the log model fits the data best ($BIC_{log} = 3060.30$ vs. $BIC_{quad} = 3069.16$). Overall GOF results and parameter estimates are listed in Tab. 12 and 13.

Table 12: Goodness-of-Fit for six models.

| | LogLik | BIC | AIC | df | $R^2$ | p [$\Delta R^2$] |
|---|---|---|---|---|---|---|
| Logistic | -1522.69 | 3074.36 | 3053.38 | 4 | - | - |
| Power | -1523.16 | 3075.31 | 3054.33 | 4 | - | - |
| Log | -1519.28 | 3060.30 | 3044.56 | 3 | 0.08 | - |
| Linear | -1522.97 | 3067.68 | 3051.94 | 3 | 0.08 | - |
| Quadratic | -1520.09 | 3069.16 | 3048.18 | 4 | 0.08 | 0.02 |
| Cubic | -1518.83 | 3073.89 | 3047.66 | 5 | 0.08 | 0.11 |

Table 13: Parameter estimates for all seven models.

| | | Estimate | Std. Error | t/z values | p |
|---|---|---|---|---|---|
| Quadratic Model | | | | | |
| | b0 | -1.228 | 0.205 | -6.004 | <.001 |
| | b1 | 4.569 | 1.083 | 4.219 | <.001 |
| | b2 | -3.326 | 1.386 | -2.400 | 0.017 |
| Two Lines Regression | | | | | |
| | b1 | 2.463 | 0.301 | 8.178 | <.001 |
| | b2 | 1.824 | 0.660 | 2.763 | 0.006 |
| | rh.breakpoint | 0.454 | . | . | . |
| Linear Model | | | | | |
| | b0 | -0.770 | 0.074 | -10.433 | <.001 |
| | b1 | 2.010 | 0.188 | 10.694 | <.001 |
| Logistic Model | | | | | |
| | b1 | 0.218 | 0.071 | 3.086 | 0.002 |
| | b2 | -5.168 | 1.273 | -4.059 | <.001 |
| | b3 | -9.087 | 1.842 | -4.934 | <.001 |
| | b4 | 0.718 | 0.014 | 52.549 | <.001 |
| Power Model | | | | | |
| | b0 | -0.774 | 0.141 | -5.487 | <.001 |
| | b1 | 2.034 | 0.199 | 10.235 | <.001 |
| | b2 | 0.989 | 0.199 | 4.966 | <.001 |
| | b3 | 0.717 | 0.014 | 52.939 | <.001 |
| Log Model | | | | | |
| | b0 | 0.723 | 0.069 | 10.513 | <.001 |
| | b1 | 0.725 | 0.066 | 11.061 | <.001 |
| Cubic Model | | | | | |
| | b0 | -1.927 | 0.486 | -3.964 | <.001 |
| | b1 | 10.843 | 4.104 | 2.642 | 0.008 |
| | b2 | -20.671 | 11.032 | -1.874 | 0.061 |
| | b3 | 14.958 | 9.439 | 1.585 | 0.113 |

*Note:*
Quadratic Model: Y = b0 + b1 * X + b2 *X^2
Two Lines Regression: Y = a + b1 * X_low + b2 * X_high + d*high
Linear Model: Y = b0 + b1 * X
Logistic Model: Y = b0 + b1 / (1 + e^(-b2 * X))
Power Model: Y = b0 + b1 * X^b2
Log Model: Y = b0 + b1 * log(X)
Cubic Model: Y = b0 + b1 * X + b2 * X^2 + b3 * X^3
t/z values contains z-values for interrupted regression, t-values for all others.

# 3 Generalizability Tests

## 3.1 LOESS Regression

For the main results shown in Figures (1, 4B, 5B and 6B), we explored the shape of the main relationships using locally estimated scatterplot smoothing (LOESS). LOESS does not require to specify any model in advance. The results in Figure 7 show again, that any u-shaped relationship is close to the edge of observed talent ratios.

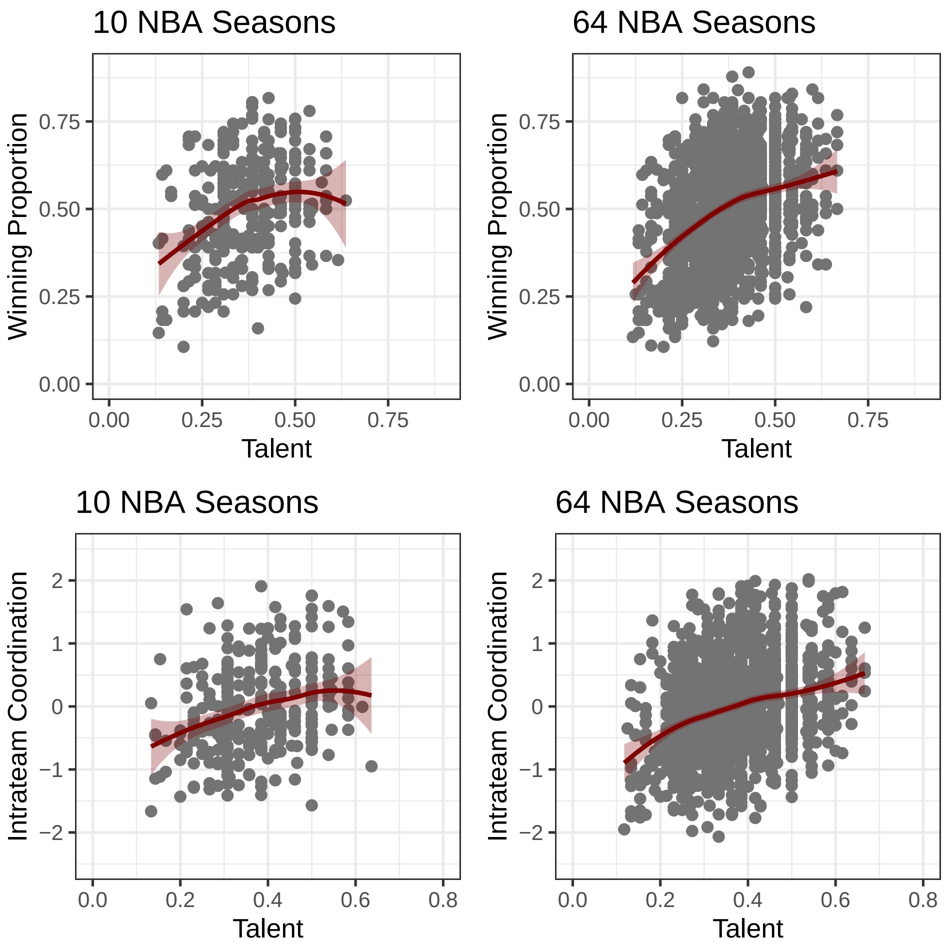


Figure 7: Loess Regression results for winning proportion (top) and intrateam coordination (bottom), 10 seasons data in left and 64 seasons data in right panel.

## 3.2 Different Talent Coding Ratios

So far, the main result of no detrimental effects of talent on performance may be due to the specific ratio used for coding talent (top 33% of EWA). In fact, Swaab et al used different ratios ("robustness checks") to check whether their main results hold. We varied the corresponding ratio as well and coded talent for ratios between 15% and 45 % of EWA in 1% steps. For each of the 31 data set, we removed extreme observations based on the same procedure as before, computed the interrupted regression and fitted all models. The analysis was repeated for both data sets (ESPN and BR) and for winning proportion and intrateam coordination. On average, excluded outliers make up less than 2.4 % of the data (max 5.7 %).

Overall, the standard approach estimates a significant (negative) quadratic term and a maximum within the observed value range in 49 out of the 124 data sets. For the interrupted regression approach we plot the $z$-statistic of the estimate of the second slope across different talent ratios (see Figure 8). The sign of $z$ corresponds to the sign of the slope with negative values indicating an inverse u-shape. The results show that for most talent ratios the slopes are either nonsignifcant or positive, suggesting no evidence of a TMT effect.

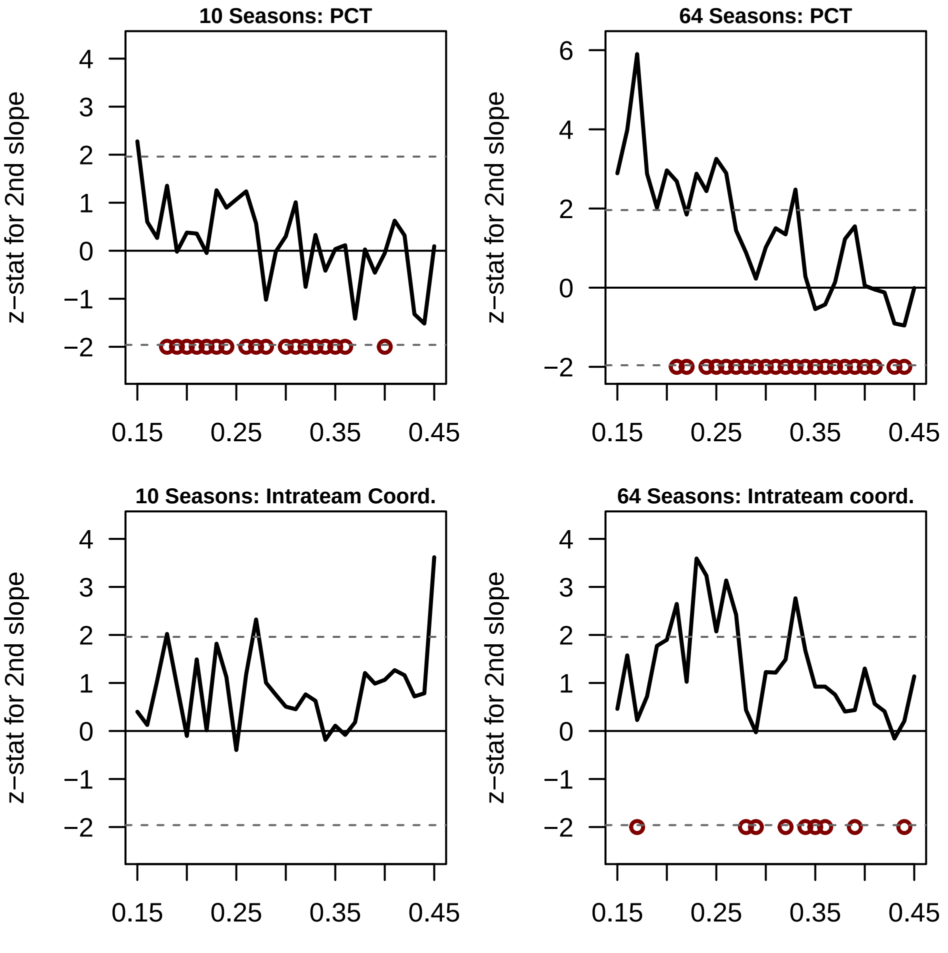


Figure 8: Z-statistics for the 2nd slope as a function of different talent ratios for winning proportion (PCT) in the top panel and intrateam coordination in the bottom panel. 10 seasons (ESPN) data on the left and 64 seasons (BR) data right. Horizontal lines mark significant z = +/-1.96 values. Red points flag cases, with a significant coefficient for the quadratic term (p <= .05) and an estimated maximum within the observed value range.

Figure 9 shows the difference in BIC between the quadratic and all other models as a function of ratios for talent coding. Positive values indicate a better GOF for the plotted over the quadratic model. As can

be seen, the log model shows the best fits across the range of ratios for winning proportion and intrateam coordination. The logistic and power models fit better than the quadratic model for winning proportion, and vice versa for intrateam coordination. However, for intrateam coordination also the linear model appears to fit the data better than the quadratic model.

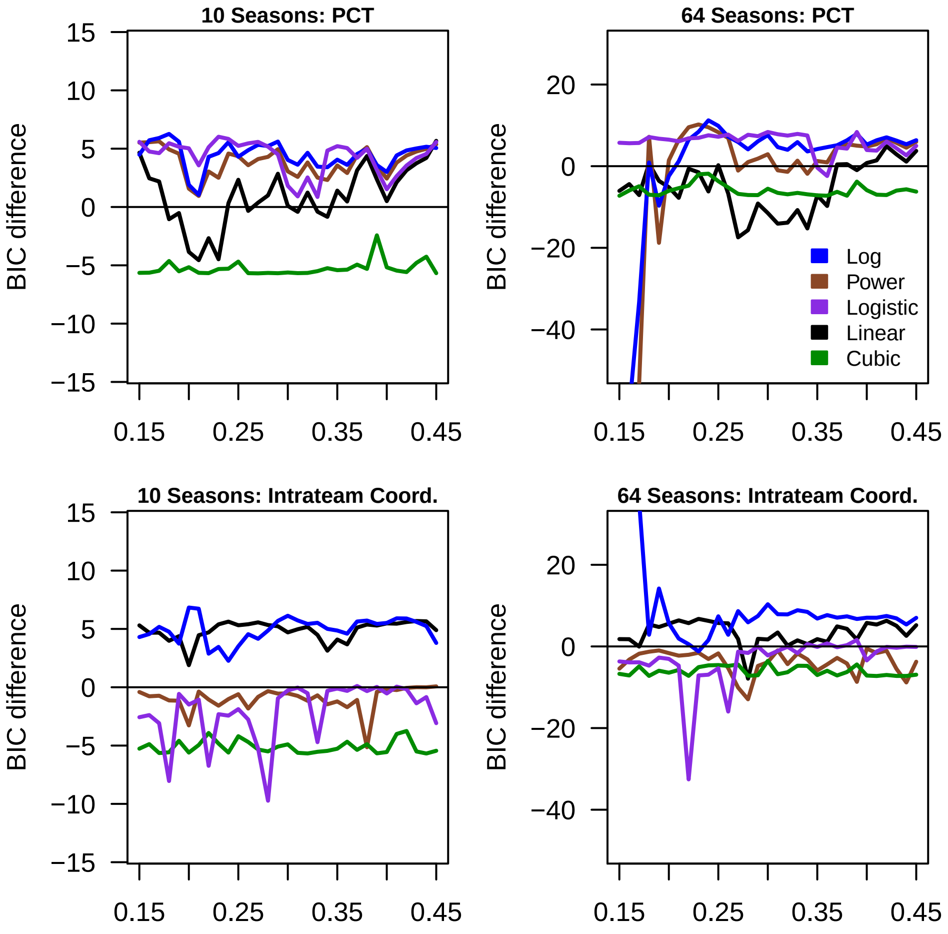


Figure 9: BIC Difference between quadratic and plotted models (quadratic - plotted) for winning proportion (PCT) in top and intrateam coordination at bottom. 10 seasons (ESPN) data in left and 64 seasons (BR) data in right panel.

## 3.3 Inclusion of Control Variables

In the main analysis in the original study the authors used "fixed-effects linear regressions of panel data, with team as the panel variable and season as the time variable" (Swaab et al., 2014, p. 1586) and four control variables: roster size, no. of games played, individual free throw percentage and lagged performance (winning proportion in previous season). In the original article, the size of the quadratic effect of talent was larger when covariates were included compared to when they were omitted. It is possible that a quadratic effect is actually present in our data and is masked by the influence of the control variables.

In order to compare our results with those reported in Table 4 of the original article, we therefore included the four covariates as well and added dummy variables for the teams to accommodate heterogeneity across teams (29 dummies: 30 teams – 1; Atlanta Hawks as the reference team). With these 33 covariates overall, we fitted (1) Model 1 with a linear effect of talent, (2) Model 2 with a quadratic effect of talent, (3) Model 3 with log(talent) and (4) Model 4 the two-lines regression. We use BIC to compare the first three models and

check the slopes for the two lines approach. We report the estimates for the 10 seasons data first, followed by the 64 seasons. TOTG, RS, FTPERC and LP refer to number of games, roster size, free throw percentage and lagged performance, estimates for dummy variables are not shown.

Table 14: Parameter estimates for the three models for 10 seasons NBA data.

| | Estimate | Std. Error | t value | Pr(>\|t\|) |
|---|---|---|---|---|
| Model 1: Talent Linear | | | | |
| (Intercept) | 0.925 | 0.257 | 3.601 | <.001 |
| TALENT | 0.238 | 0.08 | 2.963 | 0.003 |
| TOTG | -0.001 | 0.001 | -0.894 | 0.372 |
| RS | -0.026 | 0.007 | -3.69 | <.001 |
| FTPERC | -0.418 | 0.241 | -1.734 | 0.084 |
| LP | 0.424 | 0.054 | 7.901 | <.001 |
| Model 2: Talent Quadratic | | | | |
| (Intercept) | 0.657 | 0.259 | 2.536 | 0.012 |
| TALENT | 1.714 | 0.384 | 4.459 | <.001 |
| TAL2 | -1.973 | 0.503 | -3.923 | <.001 |
| TOTG | -0.001 | 0.001 | -0.996 | 0.320 |
| RS | -0.029 | 0.007 | -4.085 | <.001 |
| FTPERC | -0.365 | 0.235 | -1.554 | 0.121 |
| LP | 0.422 | 0.052 | 8.09 | <.001 |
| Model 3: Log$Talent$ | | | | |
| (Intercept) | 1.086 | 0.245 | 4.438 | <.001 |
| log(TALENT) | 0.103 | 0.027 | 3.884 | <.001 |
| TOTG | -0.001 | 0.001 | -0.842 | 0.401 |
| RS | -0.025 | 0.007 | -3.54 | <.001 |
| FTPERC | -0.413 | 0.238 | -1.733 | 0.084 |
| LP | 0.418 | 0.053 | 7.902 | <.001 |

The size of the quadratic coefficient in Model 2 is larger compared to when the control variables are omitted (see parameter estimates in section 1.2). Judged by the BIC, the quadratic model (BIC = -300.1 fits the data better than the linear model (BIC = -288.78) and the log model (BIC = -295.61). Interrupted regression estimates the breakpoint at .36. The first slope is positive and significant but the second slope is insignificant ($b_1 = 0.86$, $p < .001$; $b_2 = -0.12$, $p = .395$).

For the larger 64 seasons data the size of the quadratic coefficient (Model 2) is smaller compared to when the control variables are omitted (see corresponding estimates in section 2.2). Judged by the BIC, the model with log(talent) (BIC = -2088.59) fits the data better than the quadratic model (BIC = -2082.31) and the linear model (BIC = -2078.63). Interrupted regression estimates the breakpoint at .45. Both slopes are positive and significant ($b_1 = 0.40$, $p < .001$; $b_2 = 0.22$, $p = .025$). Overall, after inclusion of control variables the interrupted regression approach does not support the TMT hypothesis. Even at higher levels of team talent, winning proportion appears to be increasing.

Table 15: Parameter estimates for the three models for 64 seasons NBA data.

| | Estimate | Std. Error | t value | Pr(>\|t\|) |
|---|---|---|---|---|
| Model 1: Talent Linear | | | | |
| (Intercept) | 0.329 | 0.087 | 3.794 | <.001 |
| TALENT | 0.326 | 0.03 | 10.765 | <.001 |
| TOTG | 0 | 0.001 | 0.391 | 0.696 |
| RS | -0.013 | 0.002 | -5.657 | <.001 |
| FTPERC | -0.116 | 0.1 | -1.168 | 0.243 |
| LP | 0.553 | 0.02 | 26.965 | <.001 |
| Model 2: Talent Quadratic | | | | |
| (Intercept) | 0.232 | 0.091 | 2.541 | 0.011 |
| TALENT | 0.84 | 0.16 | 5.245 | <.001 |
| TAL2 | -0.669 | 0.205 | -3.268 | 0.001 |
| TOTG | 0 | 0.001 | 0.235 | 0.815 |
| RS | -0.013 | 0.002 | -5.755 | <.001 |
| FTPERC | -0.099 | 0.099 | -0.998 | 0.318 |
| LP | 0.55 | 0.02 | 26.925 | <.001 |
| Model 3: Log$Talent$ | | | | |
| (Intercept) | 0.564 | 0.087 | 6.514 | <.001 |
| log(TALENT) | 0.117 | 0.01 | 11.245 | <.001 |
| TOTG | 0 | 0.001 | 0.325 | 0.745 |
| RS | -0.013 | 0.002 | -5.673 | <.001 |
| FTPERC | -0.104 | 0.099 | -1.051 | 0.294 |
| LP | 0.55 | 0.02 | 26.904 | <.001 |

## 3.4 Winning Proportion and Intrateam Coordination in different NBA periods

For the seasons before 1979-80, baketball-reference uses adjustments to calculate the Player Efficiency Rating (PER), because some of the basic statistics are unavailable before that season (i.e. 1979-80 — debut of 3-point shot in NBA; 1977-78 — player turnovers first recorded in NBA; 1973-74 — player offensive rebounds, steals, and blocked shots first recorded in NBA). The PER measure is a crucial ingredient in the EWA measure we use here, therefore we analyzed the seasons post and pre 1979-80, separately in periods. Outliers were removed as outlined in section 1.3. First, we show the results for winning proportion (Figure 10) followed by intrateam coordination (Figure 11).

As can be seen in Figure 10, the data and the fitted quadratic model for seasons 1990-99 provide some support for the TMT effect. In seasons 1967-89 the quadratic term was positive instead of negative. However, in no period was the 2nd slope of the interrupted regression significant and negative. And, non-polynomial models (i.e. log, logistic) or the linear model fitted the data better than the quadratic or cubic models.

The corresponding results for intrateam coordination in Figure 10 show that the log model mostly provides the best approximation to data. Again, in no period was the 2nd slope of the interrupted regression significant and negative, nor was GOF of the quadratic or cubic model superior to the others. Across the total of 64 NBA seasons, it seems more plausible that true absolute talent increases with time rather than being constant. The talent-performance relationships in Figure 10 might then relate to actually higher levels of absolute talent in more recent than in the early periods. Assuming overall marginally decreasing benefits of talent, we would expect that the 2nd slope of the two-lines regression should decrease over time. Consistent with this position, the results for the 10 more recent seasons in section 1.2 show a flatter second slope than the overall results for the 64 seasons in section 2.2. However, we do not find such a consistent pattern for the 2nd slope in the analysis by periods. It would be interesting to address increasing true talent, possibly due to a more international player pool, and significant rule changes in more detail in future studies.

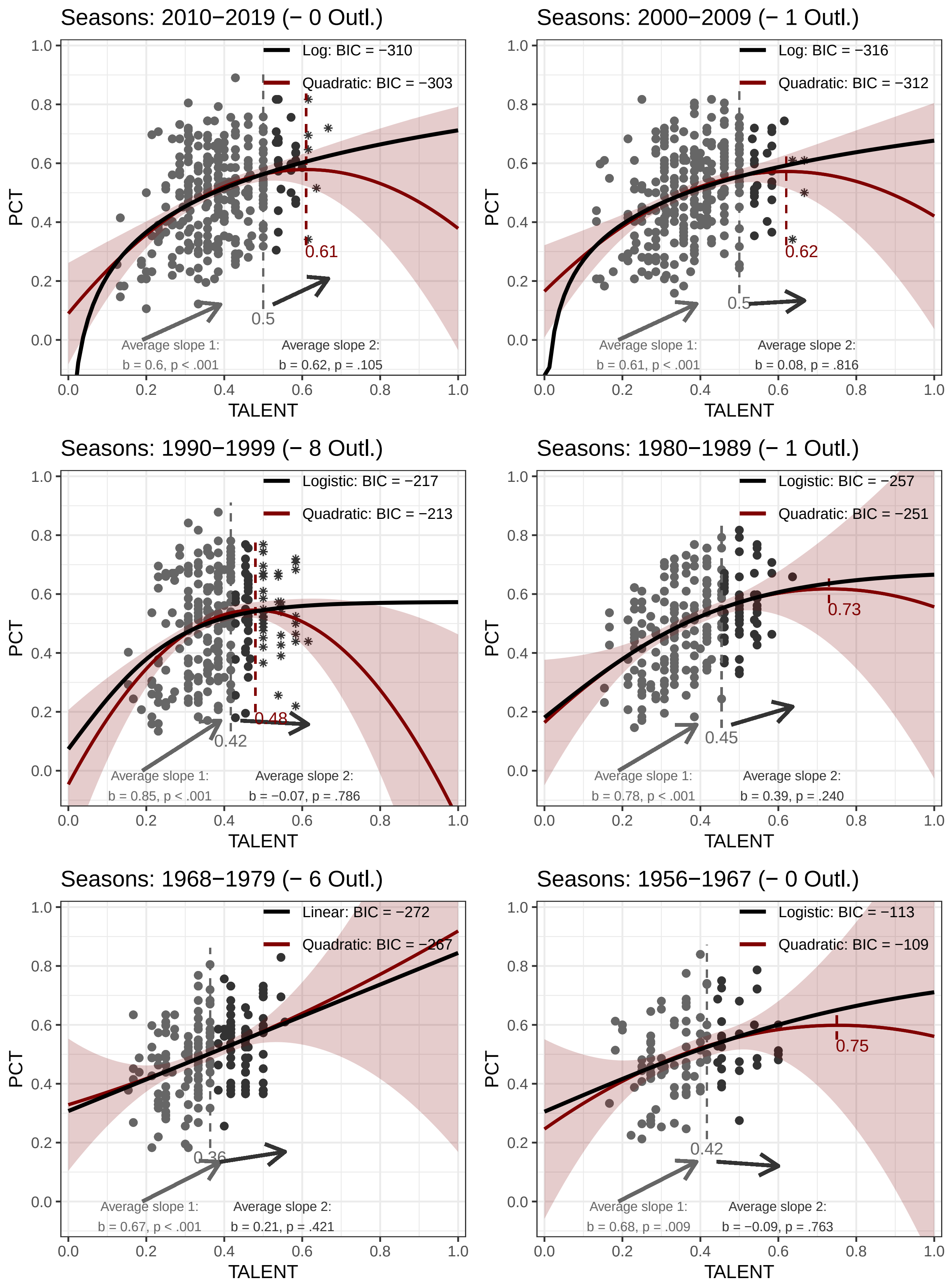


Figure 10: Winning proportion (PCT) and Talent for different NBA periods.

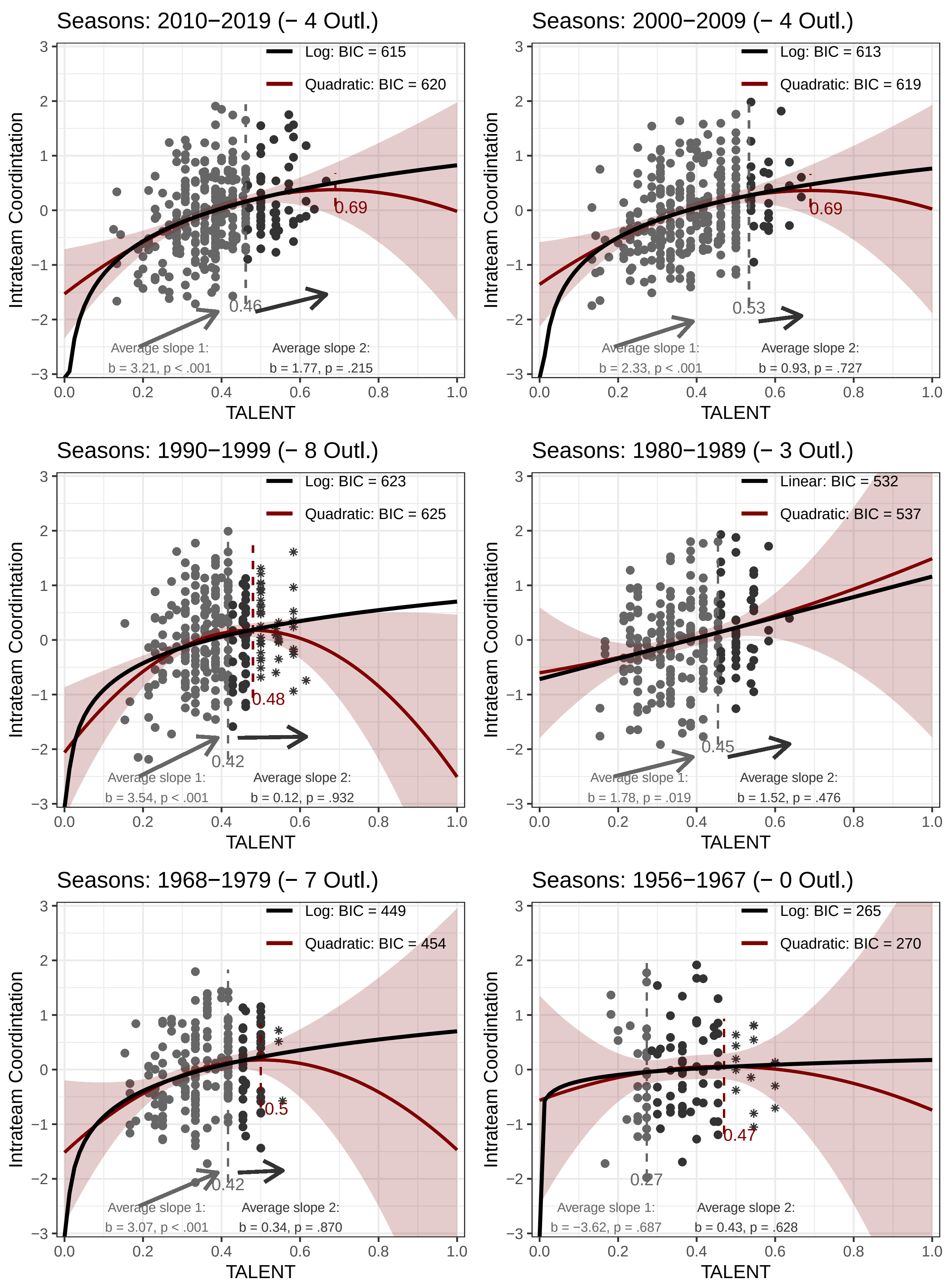


Figure 11: Intrateam Coordination and Talent for different NBA periods.

### 3.5 Other Performance Measures: WS, VORP and PER

Results for PCT and the three talent measures (Figure 12) show that the cubic and quadratic models provide the best GOF for WS and VORP. However, the 2nd slope of the interrupted regression is not significantly negative the estimated maximum from the quadratic model is beyond the observed value range. At least for WS the cubic model seems to provides some evidence in favor of the TMT effect, estimating the maximum at a talent ratio of .70 yet within the observed range (0 - .75).

For intrateam coordination (Figure 13), the quadratic model estimates maxima at the edge the observed value range for both WS (at .81) and VOPR (at .72). Beyond these maxima we find only 2 (WS) and 6 (VORP) points (0.14 % and 0.5 % of data). Again, the 2nd slope of the interrupted regression is either positive or insignificant, and the log model provides a better GOF than the quadratic model. Overall, based on different measures for team talent, we find little evidence for detrimental effects of talent on team performance.

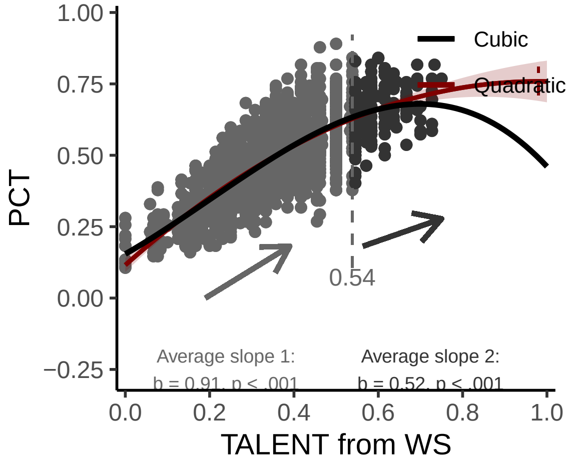


| | LogLik | BIC | AIC |
|---|---|---|---|
| **Logistic** | 1300.89 | −2580.01 | −2595.77 |
| **Power** | 1342.15 | −2662.55 | −2678.30 |
| **Log** | 1307.25 | −2592.78 | −2608.51 |
| **Linear** | 1341.84 | −2661.93 | −2677.69 |
| **Quadratic** | 1367.29 | −2705.57 | −2726.58 |
| **Cubic** | 1371.65 | −2707.04 | −2733.30 |

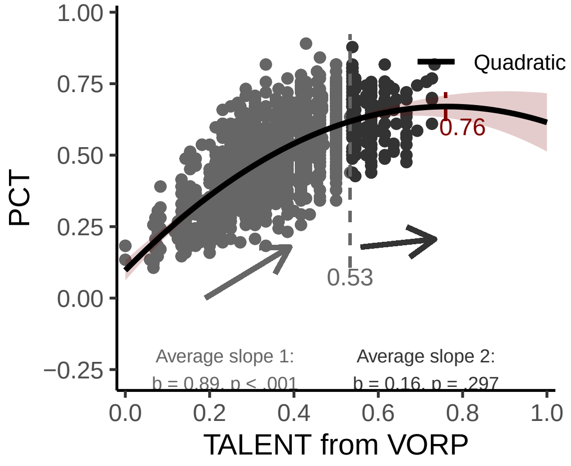


| | LogLik | BIC | AIC |
|---|---|---|---|
| **Logistic** | 984.90 | −1948.48 | −1963.80 |
| **Power** | 998.86 | −1976.40 | −1991.72 |
| **Log** | 995.07 | −1968.82 | −1984.13 |
| **Linear** | 982.17 | −1943.02 | −1958.34 |
| **Quadratic** | 1009.53 | −1990.65 | −2011.07 |
| **Cubic** | 1009.97 | −1984.40 | −2009.93 |

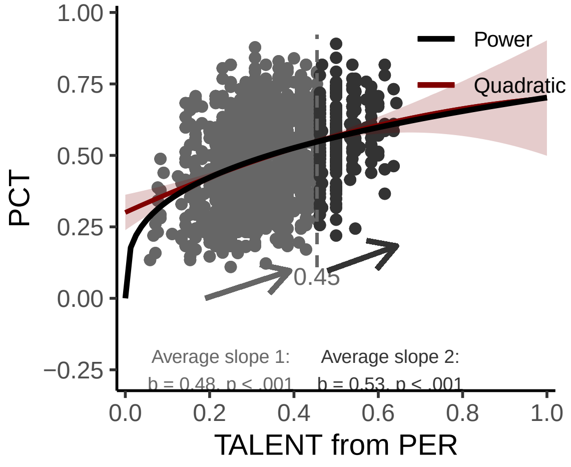


| | LogLik | BIC | AIC |
|---|---|---|---|
| **Logistic** | 762.92 | −1504.10 | −1519.85 |
| **Power** | 763.96 | −1506.17 | −1521.92 |
| **Log** | 763.01 | −1504.27 | −1520.01 |
| **Linear** | 762.55 | −1503.34 | −1519.09 |
| **Quadratic** | 763.13 | −1497.26 | −1518.26 |
| **Cubic** | 765.17 | −1494.09 | −1520.34 |

Figure 12: Winning proportion (PCT) and talent computed from three player skill measures: Win Shares (WS), Value Over Replacement Player (VORP) and Player Efficiency Rating (PER). Best fitting model in black plotted left, GOF tables on the right.

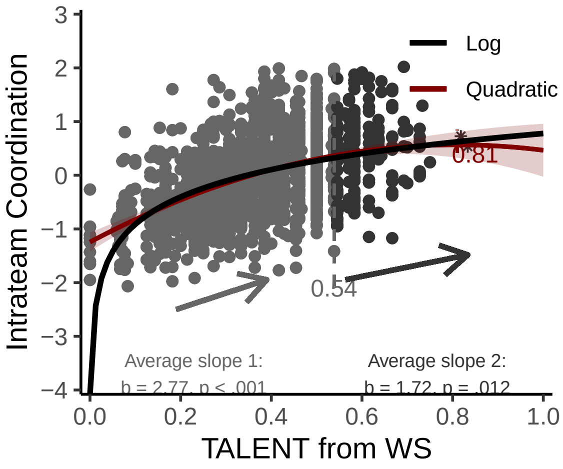


| | LogLik | BIC | AIC |
|---|---|---|---|
| **Logistic** | −1407.19 | 2843.36 | 2822.37 |
| **Power** | −1409.57 | 2848.14 | 2827.14 |
| **Log** | −1396.73 | 2815.18 | 2799.46 |
| **Linear** | −1408.04 | 2837.83 | 2822.08 |
| **Quadratic** | −1398.68 | 2826.35 | 2805.36 |
| **Cubic** | −1398.66 | 2833.56 | 2807.32 |

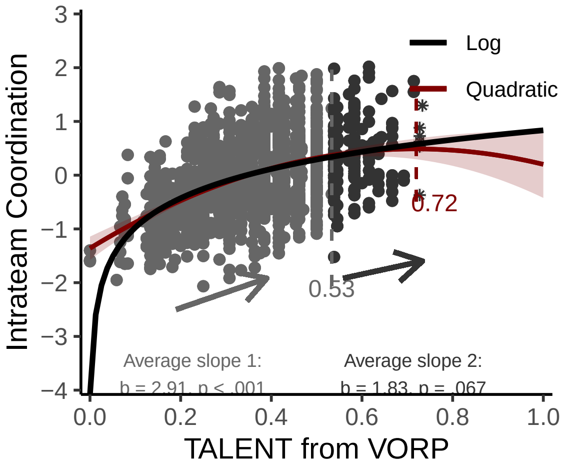


| | LogLik | BIC | AIC |
|---|---|---|---|
| **Logistic** | −1205.84 | 2440.07 | 2419.68 |
| **Power** | −1206.65 | 2441.70 | 2421.31 |
| **Log** | −1198.06 | 2417.41 | 2402.12 |
| **Linear** | −1208.87 | 2439.04 | 2423.75 |
| **Quadratic** | −1199.04 | 2426.47 | 2406.08 |
| **Cubic** | −1198.28 | 2432.04 | 2406.55 |

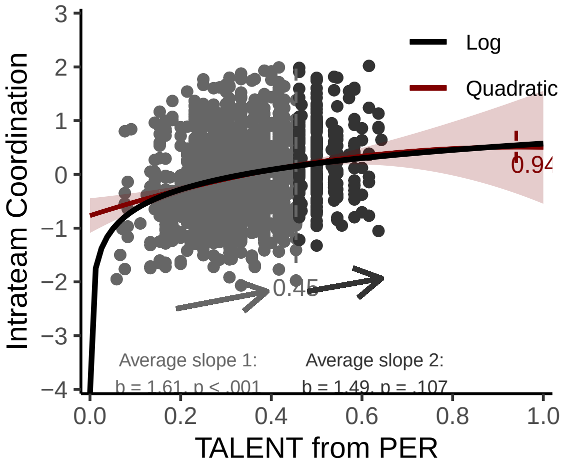


| | LogLik | BIC | AIC |
|---|---|---|---|
| **Logistic** | −1533.10 | 3095.16 | 3074.19 |
| **Power** | −1525.13 | 3079.21 | 3058.25 |
| **Log** | −1524.65 | 3071.01 | 3055.29 |
| **Linear** | −1525.80 | 3073.32 | 3057.59 |
| **Quadratic** | −1525.19 | 3079.33 | 3058.37 |
| **Cubic** | −1524.20 | 3084.60 | 3058.39 |

Figure 13: Intrateam Coordination and Talent computed from three player skill measures: Win Shares (WS), Value Over Replacement Player (VORP) and Player Efficiency Rating (PER). Best fitting model in black plotted left, GOF tables on the right.

**REFERENCES:**


Cohen, J., Cohen, P., West, S. G., & Aiken, L. S. (2003). *Applied multiple regression/correlation analysis for the behavioral sciences (3rd ed.).* Mahwah, NJ: Erlbaum.

Forster, M. R. (2000). Key concepts in model selection: Performance and generalizability. *Journal of Mathematical Psychology, 44*, 205-231.

Hollinger, J. (2005). *Pro basketball forecast: 2005-2006.* Dulles, VA: Potomac.

Roberts, S., & Pashler, H. (2000). How persuasive is a good fit? A comment on theory testing. *Psychological Review, 107*, 358-367.

Rousseeuw, P. J., & Van Zomeren, B. C. (1990). Unmasking multivariate outliers and leverage points (with discussion). *Journal of the American Statistical Association, 85*, 633–651.

Simonsohn, U. (2018). Two lines: A valid alternative to the invalid testing of U-shaped relationships with quadratic regressions. *Advances in Methods and Practices in Psychological Science, 1*, 538–555.

Swaab, R.I., Schaerer, M., Anicich, E. M., Ronay, R., & Galinsky, A.D. (2014). The Too-Much-Talent Effect: Team interdependence determines when more talent is too much or not enough. *Psychological Science, 25*, 1581-1591.

Tabachnick, B. G., & Fidell, L. S. (2014). *Using multivariate statistics (4th ed.).* New York: Pearson.

Zucchini, W. (2000). An introduction to model selection. *Journal of Mathematical Psychology, 44*, 241-261.
